# Factors Affecting Use of Preventive Tests for Cardiovascular Risk among Greeks

**DOI:** 10.3390/ijerph6102712

**Published:** 2009-10-23

**Authors:** Evelina Pappa, Nick Kontodimopoulos, Angelos A. Papadopoulos, Georgia Pallikarona, Dimitris Niakas, Yannis Tountas

**Affiliations:** 1 Hellenic Open University, Faculty of Social Sciences, Bouboulinas 57, Patras, 26222, Greece; E-Mails: nkontodi@otenet.gr (N.K.); docpapado@yahoo.gr (A.A.P.); niakas@eap.gr (D.N.); 2 “ATTIKON” University Hospital, 1 Rimini Street, Athens, 12462, Greece; 3 Centre for Health Services Research, Department of Hygiene and Epidemiology, Medical School, Athens University, 25 Alexandroupoleos Street, Athens, 11527, Greece; E-Mail: chsr@med.uoa.gr (Y.T.)

**Keywords:** preventive tests, cardiovascular disease, socio-demographic factors, self-perceived health, health risks, Greece

## Abstract

Data from a Greek national representative sample was used to investigate socio-demographic, self-perceived health, and health risk factors that determine the use of cardiovascular preventive tests (blood pressure, cholesterol and blood glucose). Chi-square and logistic regression analyses were used (p < 0.05). Older age, marriage, regular family doctor and chronic diseases increased the likelihood of receiving preventive tests, whereas low education and alcohol consumption reduced the likelihood of having these tests. The effect of obesity varied. Interventions which improve the knowledge of the poorly educated and empower the preventive role of the physicians may redress the inequalities and improve the effectiveness of preventive services utilization.

## 1. Introduction

The World Health Organisation, through the Alma-Ata declaration [1], has identified prevention as a public health priority. This has been incorporated as one of the main targets of the Greek health system, and has been addressed through the establishment of National Health System (NHS) primary care health centres. Prevention is considered as a basic component for the development of the primary health care sector and the main public schemes of primary health provision, such as NHS Health Centres and IKA (Social Insurance Fund) polyclinics—which cover about 85% of the Greek population—are responsible for providing and delivering preventive services free and equitably throughout the country.

Use of preventive services has been extensively studied in many European countries and in the U.S. The vast majority of these studies concern cancer screenings such as mammography, Pap-tests and cervical smears [2–5], whereas fewer studies concerned screenings for cardiovascular prevention such as blood pressure measurement, cholesterol screenings [4,6–10] or diabetes screening [11–14]. Socio-economic inequalities in the use of preventive services have been identified, with people better off financially reporting higher utilisation of preventive care services than those worse-off.

Prevention is not only associated with reduced morbidity and mortality, but may also result in reduced overall cost for health care systems. The appropriate use of preventive services results in early diagnosis of illness, improvement of future health, reduction of the future use of therapeutic services and of the related cost [6]. The relationship between cardiovascular prevention and the related cost—economic or other—has been studied during the last decade via a significant number of economic evaluations [15–17].

Cardiovascular disease (CVD) is a significant cause of morbidity and mortality and has a multifactorial aetiology. Studies have addressed factors that are related to the development or increase the risk of CVD such as hypertension, smoking, diabetes mellitus, dislipidaemia, obesity, alcohol consumption and physical inactivity [18–20], as well as their relation to socio-economic status [21,22].

According to the Greek National Statistical Service [23], CVD is the primary cause of mortality, and during the period 2000–2006 it accounted for 47%–52% of all recorded deaths (with a slight decline every year). Previous studies in Greece have extensively assessed the prevalence of risk factors related to the development of CVD and their relationship to socioeconomic status and lifestyle risk factors [24–27]. Contrarily, the use of preventive tests related to CVD has not yet been studied in Greece (as far as we know) and hence it is important to examine the use of preventive services for CVD in the Greek NHS, which provides access to primary and secondary health care free of charge at the point of use. The present study adds to existing research by attempting to determine the factors that affect the use of preventive screenings, in order to promote an international health policy on cardiovascular prevention.

The aim of this study was to investigate the extent to which preventive blood pressure, cholesterol and blood glucose tests are performed by the Greek population based on self-reported data. In a setting where screening rates are likely to be relatively high, we attempt to explore the factors that determine the pattern of cardiovascular preventive use, which according to the international literature are socio-demographic, access to health care, health behaviour and health need, to identify possible social disparities and to compare the results to those from other studies.

## 2. Methods

### 2.1. Sample and Data Collection

The cross-sectional study was conducted in September 2006 and involved a sample (>18 years old) residing in urban (>2,000 inhabitants) and rural (<2,000 inhabitants) areas of the country and each of the 13 geographical regions. According to the latest Population Census (2001), the survey population consisted of 8,880,924 individuals. Non-fluent Greek speakers, institutionalized subjects and those incapable of reasoning and decision-making on their own were excluded. Participants were grouped, proportionally to the Greek population, by socio-demographic characteristics, according to a three-staged sampling methodology. In the first stage, a random sample of building blocks was selected proportionally to size. In the second, households were randomly selected by systematic sampling. In the third stage an eligible participant was selected by simple random sampling in each household. In total 1,005 willing subjects, out of 1,388 initially approached (response rate 72.4%), were interviewed by trained interviewers. Participants reported information on socio-demographic characteristics, data on existing clinical conditions, health-related quality of life (measured by the SF-12), health behaviour and health services’ utilization.

### 2.2. Study Variables

In order to identify the factors that may predict the use of preventive services, we followed Andersen’s behavioural model [28], which is used to examine the use of health care services by families over a year’s time (physician ambulatory care, hospital, physician inpatient services, dental care). According to the model, utilization of health services is a function of need for care, the predisposing characteristics of the individuals and a set of factors that enable or impede the use of health services (which have a differential ability to explain use depending on what type of services are examined).

In this study, the characteristics that predispose individuals to use preventive services include gender, age, marital status and educational level (which was used as a proxy of socio-economic status), while the enabling factors that facilitate or impede use include regular source of care, private insurance and place of residence. More specifically, the independent variables were categorised by gender (male = 1, female = 0), age (ten-year groups), marital status (unmarried = 0, married = 1), education (primary = 1, secondary = 2, university = 3), residence (rural = 0, urban = 1), private insurance (yes = 1, no = 0) and access to health care measured by regular source of care (yes = 1, no = 0). Concerning the latter variable, participants responded to the question “Do you have a family doctor who advises you when necessary?”. Positive responses implied that the individuals had a regular source of care.

Need for care was addressed by two sets of proxies of health need: health status and health risks. Health status was measured by i) self-perceived health which was assessed by the Greek version of the SF-12, with higher component scores reflecting better perceived health and b) by chronic diseases, *i.e.*, people that are diagnosed having at least one chronic disease, which was contrasted to no chronic disease and used as a dichotomous variable. The SF-12 was developed as a shorter alternative to the SF-36 for use in large-scale studies and its major advantage stems from its brevity, which results in fewer burdens for researchers and respondents. The SF-12 has been validated in a representative Greek general population sample in a previous study [29].

The second set of proxies includes health risks, *i.e.*, behaviours that stem from the individual’s harmful lifestyle such as tobacco use, alcohol consumption and obesity which are known to contribute to the development of CVD. Participants were asked if they were smokers and were classified as non- versus daily/occasionally smokers. Information about alcohol consumption was based on the question “How many portions (*i.e.*, a glass of wine) of alcoholic drinks on average do you consume per week?” Respondents were classified as “up to seven glasses of wine per week” versus more. Obesity was assessed by the BMI, which is divided into three categories—normal: <24.9 bmi, overweight: 25–29.9 bmi and obese: >30 bmi.

The dependent variables were dichotomous and included use/no use of blood pressure test, cholesterol test and blood glucose test. Participants were asked when they had their last test, concerning the above preventive services, *i.e.*, “how many years has it been since your last blood pressure, cholesterol and blood glucose test?”. The majority of the users ranging from 70.4% for cholesterol to 81.3% for blood pressure had the last test within the past year from baseline (table 1).

As the focus of this study was on preventive tests for CVD risk, we excluded participants with prior conditions from the relevant tests, including 134 with hypertension, 32 with hyperlipidaemia and 62 with diabetes mellitus type I and II at baseline.

### 2.3. Statistical Analysis

Descriptive statistics have been provided and chi-square analysis was used to assess whether frequencies of preventive screenings differed across socio-demographic characteristics. Multivariable stepwise logistic regression analysis was conducted to determine the predictors of the use of preventive tests. Three logistic regression models using forward selection were applied and the exponentiation of B coefficient Exp(b) was used in order to estimate the adjusted odds ratio for each independent factor (socio-demographic, access to health care, health need proxies) with 95% confidence intervals. A supplementary analysis, as a sensitivity analysis, was carried out measuring recent use, *i.e.*, ≤2 years versus >2 years or never. The rationale behind this analysis was to minimize any recall or other measurement bias, since the participants who reported having had the tests years ago may not be accurate informants. Results were considered statistically significant when p < 0.05 and all analyses were performed using SPSS v15.0.

## 3. Results

Socio-demographic characteristics of the sample, which roughly represent the gender distribution in Greece, according to the 2001 census, and rates of preventive tests use, are provided in Table 2.

According to Table 3 there were significant differences (p < 0.05) in use of preventive tests across each socio-demographic characteristic, except for private insurance and residence. Women, more elder and those with primary education had the highest rates of use. Utilization rates appeared to be almost uniform across most of the variables except for the younger age categories, marital status and regular source of care.

### 3.1. Use of Preventive Services

Table 4 presents the logistic regression models concerning cardiovascular preventive services: blood pressure, cholesterol and blood glucose tests. According to the results, the predisposing variables gender, age and marital status affect the use of the blood pressure test. Men were about 41% less likely than women to have this test, whereas older and married people had a higher likelihood of receiving the test. The predicted odds of people aged 65+ years old having this test were almost fourteen times the odds for young people aged 18–24 years old (OR: 13.73). Individuals with a university education were almost three times more likely to receive the test (OR: 2.70) compared to individuals with only primary education, and those with a regular family doctor were 83% more likely to receive a blood pressure test (OR: 1.83) compared to those not having a regular family doctor. Only one of the variables that represent health need indicated a significant impact on the probability of receiving a blood pressure test. It was found, after controlling for the effects of the other variables, that obesity increased the use of the blood pressure test with obese individuals being almost three times more likely to have the test (OR: 2.73).

Receiving a cholesterol test depended on socio-demographic factors and health need proxies. Age was a significant determinant with individuals aged 65+ years old having an extremely high likelihood of receiving a cholesterol test compared to younger people aged 18–24 years old (OR: 10.89). Marital status and regular source of care were again significant factors with those married, and those reporting having a regular family doctor being two times more likely to have a cholesterol test than those single and those not having a regular family doctor. It was also indicated that level of education increases the probability of receiving a preventive test. Individuals with secondary education were two times more likely, than those having completed primary education, to have a cholesterol test while the likelihood increased more for university level education (OR: 2.72). Chronic diseases and alcohol consumption were the proxies of health need that predicted the use of cholesterol screening. People who suffered from at least one chronic disease (excluding those diagnosed with hyperlipidaemia) were more than two times likely (OR: 2.17) to receive the test, whereas the likelihood of those consuming more than 7 glasses of wine per week to have this test was reduced by 64% (OR: 0.358).

Concerning blood glucose tests, gender was marginally insignificant. As age increased, the likelihood of receiving a blood glucose test was extremely increased. The predicted odds, for people aged 65+ years old, of having this test were ten times the odds for young people aged 18–24 (OR: 10.07). Married, those with university education and those who have a regular family doctor had higher likelihood of receiving a blood glucose test. Considering health need proxies, chronic diseases, being overweight and alcohol consumption predicted the use of the blood glucose test. People who suffered from at least one chronic disease (excluding those diagnosed with diabetes mellitus type I and II were two times more likely (OR: 2.05) to receive the test, whereas people who consumed more than seven glasses of wine per week had a lower likelihood of having this test (OR: 0.33). Overweight individuals were 87% more likely to have a blood glucose test, whereas the results for obese individuals were not statistically significant.

The supplementary study of recent use yielded almost similar results (not shown but are available) to main use with five differences. Gender predicted recent use for a blood glucose test and did not predict recent use for a blood pressure test. Marital status did not statistically significantly affect recent use of all three tests, while chronic diseases predicted recent use of a blood pressure test (OR: 2.72, CI: 1.47–5.94). Finally, concerning the cholesterol test, results were not statistically significant for secondary education whereas BMI predicted recent use, with overweight individuals being 70% more likely to have recently had the test (OR: 1.71, CI: 1.14–2.46), but results for the obese were not statistically significant.

## 4. Discussion

In this study we attempted to identify factors that determine the use of blood pressure, cholesterol and blood glucose tests. The preventive services which were chosen are provided in a primary setting and concern cardiovascular prevention. This research, based on general population data, determined which factors among socio-demographic data, access to health care, health risks and health status are the variables that best predict the use of the above preventive services in Greece.

This study showed that the rates of blood pressure, cholesterol and blood glucose screenings were very high, ranging from 72.8% for cholesterol to 85% for the blood pressure test. The extremely high use of preventive services may be attributed to the importance of looking after one’s health or to the ease of having these tests in the Greek health care system. The latter concerns the structure of the Greek health system and especially the public sector, which is based on insurance funds with free and easy access to primary and secondary health care. The fact that these tests concern general practice and are provided by semi-urban health services, outpatient hospital departments or insurance funds which are distributed around the country, implies that people can easily receive (free of cost) these preventive tests through their insurance funds after being referred by a primary physician, outpatient hospital departments or alternatively by purchasing from the private sector.

Gender is significant predictor only for the blood pressure test, with females having a higher likelihood of receiving it. The finding is comparable to those from previous studies concerning blood pressure testing [6,7,9]. Gender is not associated with the cholesterol test, and females seem to be more likely to be screened for diabetes, as has been stated previously as well [30]. However in our study the results were marginally not significant. Additionally, use of tests follows an expected trend with age with older people being more likely to receive these services. Obviously, this is associated with the higher prevalence of cardiovascular and other chronic diseases in elderly people.

Using education as an indicator of SES, our results highlight important educational and other social inequalities in the distribution of preventive screenings. Multivariate analysis controlling for the effects of the other variables showed that people with university level education were more likely to receive blood pressure, cholesterol or blood glucose screenings than people with only primary education and blood glucose screenings in the case of secondary education. Individuals with a lower SES had a higher risk of not receiving preventive tests, and this is in line with previous studies [3,4,6–8,31], keeping in mind the fact that the concentration of health needs (*i.e.*, impaired health status) in less privileged socioeconomic groups is higher. A potential explanation may be that the higher the educational level the better the awareness or the compliance to medical advice or that the more educated were well informed [2] or had the ability to use better the available resources [4]. Our results confirmed those from a previous study, which showed that people with elementary education were less able to identify major CVD risk factors [32], which implies that interventions for dealing with inequality must be targeted at the improvement of knowledge by conducting educational programmes and campaigns for less privileged people of the society, in order to redress inequities in the distribution of the preventive screenings. The latter is related to the primary health care system in Greece which is considered to be fragmented and without continuity, generating inequality in the provision of health services and ineffectiveness. As it is stated [33], despite the several endeavours the establishment of integrated primary health care in Greece is still at its infancy.

Having a regular family doctor indicates a significant likelihood of receiving preventive tests for CVD risk, a finding that is evident in previous studies [21,34] where it was suggested that usual source of care increased the likelihood of receiving preventive services. Having a regular source of care means a well established physician-patient relationship where people have recommended services by experts and also a higher possibility for early diagnosis, good future health and quality of life. But the regular source of care is not absolutely effective. Educational differences still remain after controlling for the effect of regular source of care, implying that interventions to access to health care might only alleviate social inequalities moderately.

Two important issues must be pointed out. Firstly, the family doctor is not incorporated into the primary health care system. Usually the insured seek a family doctor in order to ensure continuity in the provision of health services by the same doctor. Secondly, due to the limited number of GPs, the family doctor is usually an internal medical doctor. Therefore the establishment of an integrated primary health care system, with the introduction of General Practitioners acting as family doctors as in the case of British NHS, may contribute to the better management of diseases and health risk factors in the community and to the development of health promotion

In terms of health need proxies, although they were consistent with the preposition that poor health is associated with increased likelihood of health care use and preventive services as well, the findings concerning the association between health risks and preventive services are not encouraging. Obesity, which is associated with increased CVD and diabetes mellitus, was not related to the use of cholesterol and diabetes tests, but it contributed significantly and positively to the use of the blood pressure test. Contrary to expectations, tobacco use did not affect the use of the preventive screenings, whereas alcohol consumption (>7 glasses/week) was associated negatively with the use of cholesterol and blood glucose screening. According to our results, those who are exposed to greater health risks were less likely to be screened, a fact that according to other studies [6,8] compromises the effectiveness of screenings for these risk factors, suggesting firstly that physicians must increase their efforts to provide preventive care to those who are inclined to harmful health behaviours, and secondly health policy interventions should support and enrich the role of prevention. The important issue is why these people did not use preventive tests. One possible explanation could be ignorance and the lack of health knowledge. A previous study in the Greek population [27] regarding the relationship between SES and cardiovascular risk factors showed that the majority (78%) of the lowest SES tertile and 23% and 21% of medium and highest tertiles respectively reported not believing that active or passive smoking may be harmful.

This study has some possible limitations. The data was based on self-reports and self-perceived health obviously involves a subjective evaluation. Self-reports for counting preventive tests may be subject to recall bias because they may overestimate the prevalence of tests, and thus may underestimate the differences in tests by social position and health risk factors. A complete health assessment requires information about the test results and whether these results were considered as normal or whether a portion of tests concerned follow-up tests due to previous abnormal results. Concerning the high rate of receiving these tests, a point that is not clear is whether these tests were periodic check-ups and were made due to systematic prevention or made on an opportunistic basis. A previous study has shown that one third of visits to family physicians involved opportunistic care [35].

## 5. Conclusions

Despite the high rates of preventive tests, social inequalities in the use of blood pressure, cholesterol and blood glucose tests still remain. Access to health care is an important factor for receiving recommended preventive tests by experts but could only merely moderate the social inequalities. Fragmentation, underdevelopment of the public primary health care and the medically-oriented Greek NHS impede the existence of an effective primary health care and produce inequalities. Problems persist with the inadequate provision of preventive tests to the most vulnerable. The establishment of an integrated primary health care system with continuity and coordination will contribute to the better management of diseases and health risk factors in the community and to the development of health promotion. Combined interventions in order to improve the knowledge of particularly the poorly educated, to empower the preventive role of the primary physicians mostly to more the vulnerable may consist an integrate approach to redress the inequalities and improve the effectiveness of the utilization of preventive services.

## Figures and Tables

**Table 1.** Time distribution of the last preventive test.

**Period**	**Blood pressure**		**Cholesterol**		**Blood glucose**	
	**N**	**%**	**N**	**%**	**N**	**%**
Within the year	664	81.3	491	70.4	514	71.6
One year ago	105	12.9	146	20.9	141	19.6
Two years ago	23	2.8	31	4.4	37	5.2
Three years ago	12	1.5	14	2.0	11	1.5
Four years+ ago	13	1.6	15	2.2	13	2.0
Don’t remember	36		35		28	
Total	853		732		744	

**Table 2.** Socio-demographic characteristics and use rates of the sample.

**Variables**	**N**	**%**
**Gender**		
Male	483	48.1
Female	522	51.9

**Age**		
18–24	115	11.4
25–34	185	18.4
35–44	180	17.9
45–54	151	15.0
55–64	150	14.9
65+	224	22.3

**Marital status**		
Unmarried	244	24.3
Married	761	75.7

**Educational Level**		
Primary	329	32.9
Secondary	491	49.1
University	180	18.0

**Family doctor**		
Yes	505	50.7
No	491	49.3
Missing	9	1.0

**Residence**		
Urban	750	74.6
Rural	255	25.4

**Private Insurance**		
Yes	112	11.4
No	867	88.6

**Blood pressure test**		
Yes	853	84.9
No	152	15.1

**Cholesterol test**		
Yes	732	72.8
No	273	27.2

**Blood glucose test**		
Yes	744	74.0
No	261	26.0

**Table 3.** Use of preventive tests according to socio-demographic characteristics.

	**Blood pressure**	**Cholesterol**	**Blood glucose**

	**N (%)**	**N (%)**	**N (%)**

**Gender**			
Male	393 (81.4)	334 (69.2)	335 (69.4)
Female	460 (88.1)	398 (76.2)	409 (78.4)
sig*	p = 0.003	p = 0.012	p = 0.001

**Age**			
18–24	69 (60)	43 (37.4)	43 (37.4)
25–34	138 (74.0)	107 (57.8)	111 (60.0)
35–44	144 (80.0)	117 (65.0)	125 (69.4)
45–54	140 (92.0)	129 (85.4)	127 (84.1)
55–64	142 (94.0)	130 (86.7)	130 (86.7)
65+	220 (98.0)	206 (92.0)	208 (92.9)
sig*	p < 0.001	p < 0.001	p < 0.001

**Marital status**			
Unmarried	159 (65.2)	116 (47.5)	196 (48.0)
Married	694 (91.2)	616 (80.9)	548 (82.4)
sig*	p < 0.001	p < 0.001	p < 0.001

**Educational level**			
Primary	301 (91.5)	264 (80.2)	270 (82.1)
Secondary	390 (79.4)	326 (66.4)	329 (67.0)
University	158 (87.7)	138 (76.7)	141 (78.3)
sig*	p < 0.001	p < 0.001	p < 0.001

**Family doctor**			
Yes	438 (89.2)	391 (79.6)	399 (66.9)
No	407 (80.6)	333 (65.9)	338 (81.3)
sig*	p < 0.001	p < 0.001	p < 0.001

**Residence**			
Yes	634 (84.5)	540 (72.0)	548 (73.1)
No	219 (85.9)	192 (75.3)	196 (76.9)
sig*	p > 0.05	p > 0.05	p > 0.05

**Private Insurance**			
Yes	96 (85.7)	85 (75.9)	88 (78.6)
No	735 (84.8)	628 (72.4)	653 (73.2)
sig*	p > 0.05	p > 0.05	p > 0.05

* 2-sided significance according to chi-square test.

**Table 4.** Logistic regression models for blood pressure, cholesterol and blood glucose tests.

	**Blood pressure**		**Cholesterol**		**Blood glucose**	

**Variables**	**OR**	**CI 95%**	**OR**	**CI 95%**	**OR**	**CI 95%**

**Gender (male)**	0.59*	0.37–0.93			0.68	0.46–1.01

**Age (18–24)**						
**25–34**	1.36	0.73–2.53	1.46	0.82–2.61	1.52	0.87–2.88
**35–44**	1.12	0.52–2.40	1.52	0.77–3.00	1.82	0.91–3.69
**45–54**	3.81**	1.41–10.31	5.77***	2.57–12.96	4.63***	2.06–10.41
**55–64**	3.16*	1.02–9.84	5.41***	2.56–12.93	4.47**	1.81–10.99
**65+**	13.73**	2.66–70.96	10.89***	4.25–27.86	10.07***	3.77–26.89

**Marital status (married)**	2.19*	1.20–4.00	1.90*	1.16–3.12	1.92*	1.14–3.22

**Education (primary)**						
**Secondary**	1.70	0.93–3.09	1.85*	1.11–3.08	1.61	0.96–2.69
**University**	2.70*	1.25–5.82	2.72**	1.47–5.04	2.81**	1.48–5.33

**Regular source of care**	1.83**	1.17–2.84	2.13***	1.48–3.05	2.26***	1.56–3.28

**PCS12**	0.96	0.92–1.00				

**Chronic diseases**			2.19**	1.31–3.66	2.05**	1.92–3.52

**BMI (normal)**						
**Overweight**	1.47	0.91–2.38			1.87*	1.23–2.84
**Obese**	2.75*	1.08–6.99			1.28	0.71–2.26

**Alcohol**			0.359*	0.14–0.92	0.33*	0.13–0.86

**R^2^**	**0.265**		**0.306**		**0.322**	

* P < 0.05;

** P < 0.01;

*** P < 0.001.
